# Histologic tau lesions and magnetic resonance imaging biomarkers differ across two progressive supranuclear palsy variants

**DOI:** 10.1093/braincomms/fcae113

**Published:** 2024-04-05

**Authors:** Francesca Orlandi, Arenn F Carlos, Farwa Ali, Heather M Clark, Joseph R Duffy, Rene L Utianski, Hugo Botha, Mary M Machulda, Yehkyoung C Stephens, Christopher G Schwarz, Matthew L Senjem, Clifford R Jack, Federica Agosta, Massimo Filippi, Dennis W Dickson, Keith A Josephs, Jennifer L Whitwell

**Affiliations:** Department of Neurology, Mayo Clinic, Rochester, MN 55905, USA; Department of Neurology and Neurophysiology, IRCCS San Raffaele University, Milan 20132, Italy; Department of Neurology, Mayo Clinic, Rochester, MN 55905, USA; Department of Neurology, Mayo Clinic, Rochester, MN 55905, USA; Department of Neurology, Mayo Clinic, Rochester, MN 55905, USA; Department of Neurology, Mayo Clinic, Rochester, MN 55905, USA; Department of Neurology, Mayo Clinic, Rochester, MN 55905, USA; Department of Neurology, Mayo Clinic, Rochester, MN 55905, USA; Department of Psychiatry and Psychology, Mayo Clinic, Rochester, MN 55905, USA; Department of Neurology, Mayo Clinic, Rochester, MN 55905, USA; Department of Radiology, Mayo Clinic, Rochester, MN 55905, USA; Department of Radiology, Mayo Clinic, Rochester, MN 55905, USA; Department of Information Technology, Mayo Clinic, Rochester, MN 55905, USA; Department of Radiology, Mayo Clinic, Rochester, MN 55905, USA; Department of Neurology and Neurophysiology, IRCCS San Raffaele University, Milan 20132, Italy; Division of Neuroscience, Neuroimaging Research Unit, Institute of Experimental Neurology, IRCCS San Raffaele Scientific Institute, Milan 20132, Italy; Department of Neurology and Neurophysiology, IRCCS San Raffaele University, Milan 20132, Italy; Division of Neuroscience, Neuroimaging Research Unit, Institute of Experimental Neurology, IRCCS San Raffaele Scientific Institute, Milan 20132, Italy; Department of Neuroscience, Mayo Clinic, Jacksonville, FL 32224, USA; Department of Neurology, Mayo Clinic, Rochester, MN 55905, USA; Department of Radiology, Mayo Clinic, Rochester, MN 55905, USA

**Keywords:** atypical PSP, neuroimaging biomarkers, tauopathy, DTI, apraxia of speech

## Abstract

Progressive supranuclear palsy is a neurodegenerative disease characterized by the deposition of four-repeat tau in neuronal and glial lesions in the brainstem, cerebellar, subcortical and cortical brain regions. There are varying clinical presentations of progressive supranuclear palsy with different neuroimaging signatures, presumed to be due to different topographical distributions and burden of tau. The classic Richardson syndrome presentation is considered a subcortical variant, whilst progressive supranuclear palsy with predominant speech and language impairment is considered a cortical variant, although the pathological underpinnings of these variants are unclear. In this case-control study, we aimed to determine whether patterns of regional tau pathology differed between these variants and whether tau burden correlated with neuroimaging. Thirty-three neuropathologically confirmed progressive supranuclear palsy patients with either the Richardson syndrome (*n* = 17) or speech/language (*n* = 16) variant and *ante-mortem* magnetic resonance imaging were included. Tau lesion burden was semi-quantitatively graded in cerebellar, brainstem, subcortical and cortical regions and combined to form neuronal and glial tau scores. Regional magnetic resonance imaging volumes were converted to *Z*-scores using 33 age- and sex-matched controls. Diffusion tensor imaging metrics, including fractional anisotropy and mean diffusivity, were calculated. Tau burden and neuroimaging metrics were compared between groups and correlated using linear regression models. Neuronal and glial tau burden were higher in motor and superior frontal cortices in the speech/language variant. In the subcortical and brainstem regions, only the glial tau burden differed, with a higher burden in globus pallidus, subthalamic nucleus, substantia nigra and red nucleus in Richardson’s syndrome. No differences were observed in the cerebellar dentate and striatum. Greater volume loss was observed in the motor cortex in the speech/language variant and in the subthalamic nucleus, red nucleus and midbrain in Richardson’s syndrome. Fractional anisotropy was lower in the midbrain and superior cerebellar peduncle in Richardson’s syndrome. Mean diffusivity was greater in the superior frontal cortex in the speech/language variant and midbrain in Richardson’s syndrome. Neuronal tau burden showed associations with volume loss, lower fractional anisotropy and higher mean diffusivity in the superior frontal cortex, although these findings did not survive correction for multiple comparisons. Results suggest that a shift in the distribution of tau, particularly neuronal tau, within the progressive supranuclear palsy network of regions is driving different clinical presentations in progressive supranuclear palsy. The possibility of different disease epicentres in these clinical variants has potential implications for the use of imaging biomarkers in progressive supranuclear palsy.

## Teaser

Orlandi *et al*. show that tau burden and MRI volume in progressive supranuclear palsy speech/language are greater in the frontal cortex but lower in subcortical/brainstem nuclei than Richardson’s syndrome. Clinical variability is, therefore, related to shifts in burden across a network of regions.

## Graphical Abstract

**Graphical Abstract:**
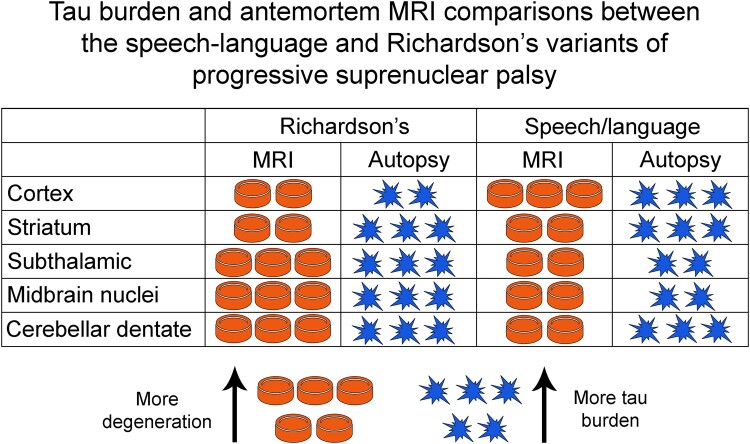

## Introduction

Progressive supranuclear palsy (PSP) is a neurodegenerative disorder caused by the accumulation of four-repeat (4R) tau protein in neuronal and glial cells.^1^ Neuronal lesions include globose-shaped neurofibrillary tangles located in the cytoplasm and dense neuropil threads involving neuronal processes. Glial lesions include comma-like coiled bodies within oligodendrocytes and star-shaped tufted astrocytes.^2,3^ Different topographic patterns of tau burden underlie multiple PSP pathological subtypes that manifest as different PSP clinical variants.^4^ Clinically, the classical form of PSP is known as Richardson syndrome (PSP-RS) and presents with early postural instability resulting in falls, vertical supranuclear gaze palsy, early levodopa unresponsive parkinsonian syndrome and cognitive involvement.^5^ Over the years, several authors have reported different clinical variants associated with PSP pathology^6-11^ and many of these clinical variants were recognized in the most recent PSP clinical diagnostic criteria.^12^ One such variant is PSP with a predominant speech and language disorder (PSP-SL). These patients present with motor speech or language disturbances,^10,13,14^ typically diagnosed as either primary progressive apraxia of speech^15^ or non-fluent variant of primary progressive aphasia,^16^ and, over time, may progress to develop parkinsonism and ocular motor impairments.^17^ In terms of pathology, the ‘typical’ distribution of tau pathology in PSP-RS is concentrated in the basal ganglia, diencephalon and brainstem nuclei (subthalamic nucleus and substantia nigra).^18^ When pathology defies this pattern with a more widespread neocortical involvement, the term ‘atypical’ PSP is utilized.^4,10,18^ We have previously observed atypical PSP pathology in four PSP-SL cases, suggesting that a more neocortical distribution of tau pathology underlies this clinical presentation.^10^

Previous MRI research has mostly focused on PSP-RS,^19-24^ which shows atrophy of the midbrain, subcortical grey matter, superior cerebellar peduncles and precentral cortex. Diffusion tensor imaging (DTI) studies have also demonstrated striking degeneration of white matter tracts, including the superior cerebellar peduncles, the body of the corpus callosum and some association tracts.^25,26^ In contrast, PSP-SL patients have predominant atrophy in premotor and motor cortices with less involvement of the midbrain and subthalamic nucleus compared with PSP-RS.^17,24^ PSP-RS also has greater DTI abnormalities in the superior cerebellar peduncle than PSP-SL, whilst PSP-SL shows greater degeneration of the body and genu of the corpus callosum, internal capsule, external capsule and superior longitudinal fasciculus.^25^ However, approximately a third of patients clinically diagnosed with PSP-SL do not have PSP at autopsy^27^ and the extent to which neuroimaging findings mirror the underlying neuropathological substrates remains poorly understood. Moreover, because of the persistent lack of validated tau PET radiotracers with high 4R tau-binding specificity, reliable *in vivo* estimation of the underlying true pathology is still challenging. To our knowledge, only a couple of studies have assessed the correlation between neuropathology and neuroimaging in 4R tauopathies,^28,29^ and none have focused on the relationship between specific tau lesion subtypes and neuroimaging in PSP-SL.

Our primary aim was to determine whether PSP-RS and PSP-SL have different regional distributions or burden of neuronal and glial tau lesions in selected neocortical, subcortical, brainstem and cerebellar regions of interest (ROIs), with an in-depth description at the level of single lesion subtypes. Our secondary aim was to investigate differences in neuroimaging features between the two variants and to further identify correlations between pathology and imaging metrics. We hypothesized that a shift of tau pathology from subcortical to neocortical structures would be present in PSP-SL and that increased regional tau burden would correlate with more volume loss and white matter structural damage, but that the strength of these relationships would vary depending on the type of cellular lesion (neuronal versus glial). Understanding these relationships may provide crucial information on the pathophysiology of these PSP variants and help determine the value of neuroimaging metrics as biomarkers of tau pathology for future clinical treatment trials in PSP.

## Materials and methods

### Participants

All patients were recruited by the Neurodegenerative Research Group at Mayo Clinic in Rochester, MN, USA between April 2010 and August 2020, died and received an autopsy diagnosis of PSP. All patients underwent serial neurological examinations and 3T MRI scans during life. To be included in the present study, patients must have had a clinical diagnosis of either PSP-RS or PSP-SL before death and at least one available *ante-mortem* MRI. A clinical diagnosis of PSP variant was made according to the 2017 Movement Disorder Society Criteria,^12^ operationalized as previously described.^17^ All patients with PSP-SL had been followed longitudinally in a study focused on primary progressive apraxia of speech/non-fluent variant of primary progressive aphasia and all had undergone detailed speech and language evaluations by a speech–language pathologist. Patients were included if they met the criteria for either suggestive or possible PSP-SL and died with a primary PSP pathological diagnosis. Disease onset in the PSP-SL patients was considered as the onset of speech and language complaints, whilst disease onset in the PSP-RS patients was considered as the onset of the first PSP features. In the case of multiple scans, the scan closest to death was used. Consequently, our final cohort consisted of 33 autopsy-confirmed PSP patients, 17 probable PSP-RS and 16 PSP-SL. The study was approved by the Mayo Clinic institutional review board. All patients or their proxies provided written informed consent and consented to the brain donation.

### Clinical assessment

The neurological evaluations included the administration of different standardized and validated tests, including the Montreal Cognitive Assessment^30^ to test for global cognitive function, the Frontal Assessment Battery^31^ for executive functions, the Movement Disorder Society-sponsored revision of the Unified Parkinson’s Disease Rating Scale III^32^ for parkinsonism and gait abnormalities and the PSP Rating Scale^33^ for overall disease severity and disability. PSP Rating Scale subscores for mentation, gait and midline and oculomotor function were assessed. As a further means to assess oculomotor function and determine the presence of supranuclear gaze palsy, the PSP Saccadic Impairment Scale (PSIS)^34^ was administered.

All PSP-SL patients (*n* = 16) and a subset of PSP-RS patients (*n* = 8) additionally underwent comprehensive speech and language evaluations. The Northwestern Anagram Test^35^ was used to evaluate syntax competence, and the Boston Naming Test^36^ was used to test confrontation naming. Speech-related motor functions were assessed with the Apraxia of Speech Rating Score version 3^37^ and the Motor Speech Disorder Severity Rating.^38^ Diagnoses regarding the presence, nature and severity of apraxia of speech and aphasia were made by consensus between at least two board-certified speech–language pathologists. When speech and language data were not available at the last visit before death, we used the last available assessment from a previous visit.

### Neuropathological evaluations and tau semi-quantitative scoring

Following autopsy, the neuropathological evaluation was systematically conducted on the left fixed hemibrain, except for three cases whereby the right (*n* = 1), bilateral (*n* = 1) or unspecified (*n* = 1) hemisphere was sampled. Brain tissue was formalin fixed, paraffin-embedded and processed to yield 5- to 8-*µ*m slices used for staining with haematoxylin and eosin, thioflavin-S and Gallyas silver stain. Ten pathological ROIs were obtained as previously reported.^28^ These ROIs were selected from four different macro-regions of the brain: cortical, subcortical, brainstem and cerebellar. From the cortical region, sections of the superior frontal gyrus and precentral gyrus (motor cortex) were derived from coronal cuts at the level of anterior hippocampus and the posterior frontal cortex, respectively. From the subcortical region, sections of the ventral thalamus and subthalamic nucleus were identified through a coronal cut caudal to the posterior edge of the mammillary bodies, whilst sections of the striatum and globus pallidus were obtained from a cut at the level of the anterior commissure. From the brainstem, sections showing the midbrain tectum, substantia nigra and red nucleus were obtained through a transverse cut made at the level of the superior colliculus. For the cerebellar dentate nucleus, a transverse cut of the cerebellum was performed. The presence of 4R tau pathology was assessed using slides immunostained with anti-phospho-tau monoclonal antibody (CP13; 1:1000; mouse IgG1 to phosphoserine 202, from the late Dr Peter Davis, Feinstein Institute, Long Island, NY, USA). The diagnosis of PSP pathology was made in all cases according to published criteria^3^ by an experienced neuropathologist (D.W.D.). Differentiation between typical and atypical PSP pathology^10^ was also reported, with atypical cases grossly displaying more severe cortical than subcortical/brainstem pathology. Co-pathologies were also assessed according to previously reported guidelines for the diagnosis of Alzheimer’s disease neuropathological changes^39^ [Thal amyloid-β phases,^40^ Braak neurofibrillary tangle stages^41^ and Consortium to Establish a Registry for Alzheimer’s disease neuritic plaque score^42^] Lewy Body disease,^43^ argyrophilic grain disease^44^ and aging-related tau astrogliopathy.^45^ The presence of cerebral amyloid angiopathy and arteriolosclerosis was also assessed using slides stained with haematoxylin and eosin.

For each of the 10 above-mentioned pathological ROIs, a semi-quantitative assessment of the 4R tau lesion burden was performed, as previously described.^28^ The four main tau lesions of interest were as follows: neurofibrillary tangles/pre-tangles and neuropil threads (both predominantly associated with neuronal cells) and tufted astrocytes and coiled bodies (both associated with glial cells). In each region, neurofibrillary tangles, coiled bodies and tufted astrocytes were graded on a scale from 0 to 3 based on the number of lesions observed per ×200 magnification visual field: 0 = no lesions, 1 = mild (1–3 lesions), 2 = moderate (4–6 lesions) and 3 = severe (>7 lesions). Neuropil threads were graded 0–3 for absent, low, intermediate and high density of lesions. Composite scores for neuronal and glial tau were obtained by summing single lesion counts so that the maximum obtainable score was six. The Kovacs staging scheme^46^ was also applied using the semi-quantitative lesion scores (scores from the cerebellar white matter were utilized but ratings for the occipital lobe were not available).

### Volumetric MRI and DTI metrics

All patients underwent T_1_-weighted volumetric MRI with either GE (*n* = 21) or Siemens (*n* = 12) scanners. The imaging protocol included a 3D Magnetization Prepared Rapid Acquisition Gradient-Echo sequence. For GE scanners, parameters were repetition time/echo time/inversion time 2300/3/900 ms; flip angle 8°; 26-cm field of view; 256 × 256 in-plane matrix with a phase field of view of 0.94; slice thickness of 1.2 mm; and in-plane resolution of 1 mm. For Siemens scanners, parameters were repetition time/echo time/inversion time 2300/3.14/945 ms, flip angle 9° and 0.8-mm isotropic resolution in a 320 × 300 matrix.

We selected imaging ROIs that best corresponded to our pathological ROIs except for the midbrain, whose overall volume was compared against the average of tau burden calculated from the red nucleus, substantia nigra and midbrain tectum. Processing of the Magnetization Prepared Rapid Acquisition Gradient-Echo consisted of Unified Segmentation in SPM12 and scan normalization to the Mayo Clinic Adult Lifespan Template^47^ using ANTs.^48^ The ANTs mapping from each Mayo Clinic Adult Lifespan Template to patient Magnetization Prepared Rapid Acquisition Gradient-Echo was applied to the ROI atlases to calculate regional values for each patient. Volumes of the superior frontal cortex, precentral cortex, thalamus, striatum (caudate plus putamen) and globus pallidus were calculated using the Mayo Clinic Adult Lifespan Template atlas. For the substantia nigra, subthalamic nucleus and red nucleus, the Deep Brain Stimulation Intrinsic Template atlas^49^ was used to determine volumes. Volumes of the midbrain and cerebellar dentate nucleus were derived from in-house atlases, as previously described.^50^ Total intracranial volumes were also calculated to allow correction for differences in head size. All volumes were converted to *Z*-scores, indicating the overall degree of abnormality using a cohort of controls matched 1:1 by age, sex, education and MRI manufacturer, that had undergone identical neuroimaging protocols as the main study patients (Supplementary Table 1). The control cohort was divided by scanner manufacturer, with matched GE controls used to calculate *Z*-scores for GE patients and matched Siemens controls used to calculate *Z*-scores for Siemens patients. Hence, the analysis used *Z*-scores rather than raw volumes from different manufacturers to minimize any systematic bias.

For DTI, we analysed the 21 patients who underwent MRI on GE scanners. The DTI acquisition consisted of a single shot echo-planar imaging pulse sequence (acquisition matrix 128 × 128 in axial plane; approximate echo time=64 ms; repetition time=10 s; field of view= 35 cm; phase field of view= 0.66; 41 diffusion encoding directions and five non-diffusion weighted (*b* = 0) T_2_ images; and slice thickness 2.7 mm), as previously described.^28^ The obtained diffusor tensors were used for the calculation of mean diffusivity (MD) and fractional anisotropy (FA) images in dipy.^51^ The John Hopkins University (JHU)^52^ atlas was used to measure regional FA and MD, as previously described.^28^ To match our pathological ROIs, we selected DTI metrics (both FA and MD) from the superior frontal white matter, precentral white matter, midbrain and superior cerebellar peduncle. MD was additionally measured in grey matter structures like the globus pallidus, thalamus and striatum (caudate and putamen). Both FA and MD calculated from the superior cerebellar peduncle were compared with tau lesion scores in the red nucleus and cerebellar dentate.

All imaging variables were measured from the same hemisphere on which the neuropathological evaluation was performed. An exception was two cases (both PSP-SL) where either both hemispheres were sampled or the laterality of the hemisphere sampled was unknown, in which case we took an average of left and right volumes or DTI metrics.

### Statistical analysis

Categorical variables were reported as counts and percentages, whilst continuous variables were reported as medians and interquartile ranges. Comparison of data between the two groups was performed using chi-square test for categorical variables and Wilcoxon ranked-sum test for continuous variables, with the threshold for significance set at *P* < 0.05. Area under the receiver operator characteristic curve values are provided as a measure of effect size, along with 95% confidence intervals. To determine correlations between histologic lesions and imaging metrics, we fit single-region linear regression models with *Z*-score converted volumes and log-transformed DTI metrics (MD and FA) as the response and the semi-quantitative tau burden as the predictors of interest, with age at death and time from MRI scan to death as covariates. Two different models were used. In the first model, tau burden (neuronal or glial) was used as a predictor of changes in volumes, FA or MD. To test whether tau burden had a significant effect on imaging metrics, we also ran an ANOVA test to compare the models with and without tau burden (i.e. with age at death and time from MRI scan to death only as predictors). In the first model, PSP variant was added to determine whether different tau effects varied by PSP variant. Regression coefficients were obtained separately for both PSP-RS and PSP-SL. In the second model, an interaction term between PSP variant and tau burden was included to assess significant differences in effect sizes between the two PSP variants. For the models using DTI metrics, we back-transformed the regression coefficients obtained using the formula (exp(beta)−1)×100, to interpret effect of predictors as percentage change. We evaluated potential non-linearity in the neuronal and glial tau burden associations using restricted cubic splines with three knots.^53^ All statistical analyses were performed with R, version 4.2.2. Results were considered significant at a *P*-value of <0.05, and all results were also corrected for multiple comparisons using the false discovery rate (FDR).

## Results

### Characteristics of patients

Patient characteristics are summarized in Table 1. The PSP-RS patients were younger at both onset and death and were slightly less educated than the PSP-SL patients. Both disease duration and time from onset to neurological evaluation were shorter in PSP-RS. Consistently, PSP-RS also had a shorter time from onset to MRI and time from MRI to death.

**Table 1:** Patient characteristics

	PSP-RS (*n* = 17)	PSP-SL (*n* = 16)	*P*-value
**Demographics**			
No, females (%)	6 (35%)	6 (38%)	0.90
Ethnicity (White, %)	16 (94%)	14 (88%)	0.61
Education (years)	14.9 [12–20]	15 [13.5–16]	**0**.**04**
Handedness: right, left, ambidextrous	15 (88%), 1 (6%), 1 (6%)	13 (81%), 2 (13%), 1 (6%)	0.79
Age at onset	65 [59–68]	70.5 [65.5–73]	**0**.**01**
Age at death	69 [63–75]	82 [76–83]	**<0.001**
Disease duration	5.72 [5.23–7.28]	10.4 [9.27–11.2]	**<0.001**
Time from disease onset to neurological evaluation, *y*	4.29 [3.33–5.71]	8.13 [6.19–9.36]	**<0**.**001**
Time from neurological evaluation to death, *y*	1.26 [0.91–2.43]	2.61 [1.62–3.07]	0.05
**Clinical features** ^a^			
MoCA^b^	22 [16–23]	22 [18–24]	0.66
MDS-UPDRS III (/132)	59 [47–72]	59.5 [25.8–74.5]	0.63
PSP Rating Scale (/100)	57 [46–67]	46.5 [25–58.8]	0.07
PSP rating scale—mentation (/16)	5 [3–8]	5 [3–6.5]	0.83
PSP rating scale—gait and midline (/20)	16 [13–20]	11.5 [3.75–16.2]	**0**.**03**
PSP rating scale—ocular motor (/16)	11 [10–14]	8 [5–11]	**0**.**005**
PSIS (/5)	4 [4–4]	2 [1.75– 3]	**<0**.**001**
FAB (/18)	13 [10–15]	12 [9–14]	0.38
**Speech and langauge** ^c^			
NAT (/10)	***-***	7 [5–9]	-
BNT (/15)	14 [14–14]	14 [13–15]	>0.99
ASRS v3 (/52)	3.5 [2–5]	32 [28.2–35.5]	**<0.001**
MSD severity score (/10)	6 [6–7]	4 [2–5]	**0**.**003**
**Neuromaging**			
Age at last MRI, *y*	68.6 [62.1–74.2]	77.7 [73.9–81.7]	**<0**.**001**
Time from onset to last MRI, *y*	4.29 [3.34–5.71]	7.73 [6.18–9.36]	**<0**.**001**
Time from last MRI to death, *y*	1.26 [0.91–2.43]	2.61 [2.08–3.07]	**0**.**02**
**Neuropathology**			
Post-mortem interval, hours^d^	17 (10, 18)	12 (9, 38)	0.90
PSP pathology: typical (%), atypical (%)	16 (94%), 1 (6%)	6 (37.5%), 10 (62.5%)	**<0**.**001**
Thal phase: 0, 1, 2, 3	9 (53%), -, 4 (23.5%), 4 (23.5%)	5 (31.3%), 1 (6%), 5 (31.3%), 5 (31.3%)	0.62
Braak stage: Absent, I, II, III, IV, V, VI	2 (12%), 4 (23.5%), 4 (23.5%), 5 (29%), 2 (12%), -, -	1 (6.25%), 1 (6.25%), 1 (6.25%), 9 (53%), 3 (19%), 1 (6.25%), -	0.29
Lewy body: present	1/4 (25%)	-	0.40
ARTAG status: positive	4/16 (25%)	9/15 (60%)	0.05
AGD status: positive	2/15 (13%)	6/14 (43%)	0.11
CAA positive, *n* (%)	7 (41%)	7 (44%)	0.88
Arteriolosclerosis: none, mild, moderate, severe	5/15 (33%), 6/15(40%), 4/15 (27%), -	3/16 (19%), 5/16 (31%), 4/16 (25%), 4/16 (25%)	0.21

Data are reported as median [Q1, Q3] or count (%).

Threshold of significance was set at 0.05. Significant *P*-values are reported in bold.

Speech and language assessment at last visit before death was available for a limited set of patients. Here, we report data from last available evaluation.

AGD, argyrophilic grain disease; ARTAG, age-related tau astrogliopathy; ASRS v3, Apraxia of Speech Rating Scale version 3; BNT, Boston Naming Test; CAA, cerebral amyloid angiopathy; FAB, Frontal Assessment Battery; MDS-UPDRS III, Movement Disorders Society-sponsored revision of the Unified Parkinson’s Disease Rating Scale; MoCA, Montreal Cognitive Assessment; MSD Severity Score, Motor Speech disorder severity score; NAT, Northwestern Anagram Test; PSP, progressive supranuclear palsy.

^a^All clinical data are from the last available neurological evaluation.

^b^MoCA scores were converted from MMSE for seven patients (see main text).

^c^Only 8/17 PSP-RS patients had available data.

^d^Post-mortem interval (time between death and brain removal) was available for 17 PSP-RS and 16 PSP-SL patients.

At the last available clinical evaluation before death, the PSP-SL patients had more motor speech impairment as indexed by higher ASRS scores and lower Motor Speech Disorder Severity Ratings. The two groups did not differ in measures of overall cognition and mentation. PSP-RS showed more impaired balance and gait and oculomotor functions on the PSP Rating Scale than PSP-SL, and the PSIS was also higher in PSP-RS. On further evaluation of the PSIS scores (Supplementary Table 2), one PSP-SL patient never developed any signs of ocular motor dysfunction (PSIS = 0) and hence only met the criteria for suggestive of PSP-SL.^12^ Nine patients had moderate oculomotor dysfunction with slowing of vertical saccades (PSIS = 1 or 2), and most of these patients were PSP-SL. Twenty-three patients had complete vertical supranuclear gaze palsy (PSIS = 3–5) with more frequent diagnoses of PSP-RS. Of the PSP-SL patients, 44% had developed postural instability or falls, and 88% had developed akinesia, by the last evaluation. However, only three would have met the criteria for PSP-RS,^12^ given that postural instability/falls must be observed within three years of onset.^12^

Neuropathological assessment at autopsy revealed that typical PSP pathology was more frequently associated with PSP-RS, whilst atypical PSP pathology was related to PSP-SL. Frequencies of co-pathologies were comparable across the two groups. Of the 33 cases, 23 had a Kovacs stage of at least 4 and the remaining nine were not able to be classified due to the presence of moderate frontal/cerebellar dentate lesions in the presence of only mild lesions in globus pallidus or striatum (four PSP-RS and five PSP-SL).

### Histologic differences across PSP-RS and PSP-SL

#### Neuronal and glial tau distribution patterns

Differences in composite neuronal and glial tau lesion scores between the two PSP variants are displayed in Table 2. PSP-SL showed a higher burden of neuronal tau in both the motor cortex and superior frontal cortex. All other ROIs from the subcortical, brainstem and cerebellar regions had similar levels of moderate–severe neuronal tau pathology in both groups. Glial tau pathology was more variable. PSP-SL had higher levels of glial tau in both the motor cortex and superior frontal cortex than PSP-RS, whilst PSP-RS showed higher levels of glial tau in subthalamic nucleus. PSP-RS also showed higher levels of glial tau in the globus pallidus, substantia nigra and red nucleus, although these findings did not survive correction for multiple comparisons. There were no differences in neuronal or glial tau lesion scores in the striatum, midbrain or cerebellar dentate.

**Table 2:** Neuronal and glial tau lesion count comparison across groups

	PSP-RS (17)	PSP-SL (16)	AUROC (95% CI)	*P*-value
**Neuronal tau**				
Motor cortex	4 [3–4]	5.5 [5–6]	0.82 (0.62, 0.92)	**0**.002*
Superior frontal cortex	4 [3–4]	5 [5–5]	0.75 (0.55, 0.87)	**0**.006*
Striatum	4 [3–5]	4 [3–5]	0.52 (0.34, 0.70)	0.84
Globus pallidus	4 [4–5]	3.5 [3–5]	0.65 (0.45, 0.80)	0.15
Ventral thalamus	5 [5–6]	6 [5–6]	0.55 (0.36, 0.73)	0.43
Subthalamic nucleus	6 [5–6]	5 [5–6]	0.62 (0.42, 0.78)	0.19
Substantia nigra	5 [4–5]	5 [4–5.25]	0.51 (0.32, 0.69)	0.97
Red nucleus	5 [5–6]	6 [4–6]	0.53 (0.34, 0.71)	0.74
Midbrain tectum	6 [5–6]	5 [4.5–5.5]	0.69 (0.49, 0.83)	0.05
Cerebellar dentate	4 [3– 5]	5 [3.75–6]	0.64 (0.44, 0.79)	0.18
**Glial tau**				
Motor cortex	4 [3–4]	5 [4.75–6]	0.76 (0.56, 0.88)	**0**.01*
Superior frontal cortex	4 [3–4]	5 [5–5]	0.77 (0.57, 0.89)	**0**.006*
Striatum	5 [4–6]	5 [4–5.25]	0.57 (0.37, 0.74)	0.51
Globus Pallidus	4 [3–5]	2 [1–3.25]	0.72 (0.52, 0.85)	**0**.**03**
Ventral thalamus	4 [3–5]	2 [2–4]	0.70 (0.49, 0.84)	**0**.**05**
Subthalamic nucleus	3 [3–5]	2 [1–3.25]	0.77 (0.57, 0.89)	**0**.007*
Substantia nigra	2 [1–4]	1 [1–2]	0.70 (0.50, 0.84)	**0**.**04**
Red nucleus	4 [3–5]	3 [1.75–4]	0.71 (0.51, 0.85)	**0**.**04**
Midbrain tectum	6 [5–6]	4 [3.5–5]	0.68 (0.47, 0.82)	0.08
Cerebellar dentate	1 [1–1]	1 [0.75–1]	0.57 (0.38, 0.74)	0.42

Data are reported as median [Q1, Q3] or count (%).

Threshold of significance was set at 0.05. Significant *P*-values are reported in bold.

AUROC, area under the receiver operator characteristic curve.

^*^ *P*-values significant after FDR correction.

#### Single tau lesion subtypes distribution patterns

**Figure 1:**
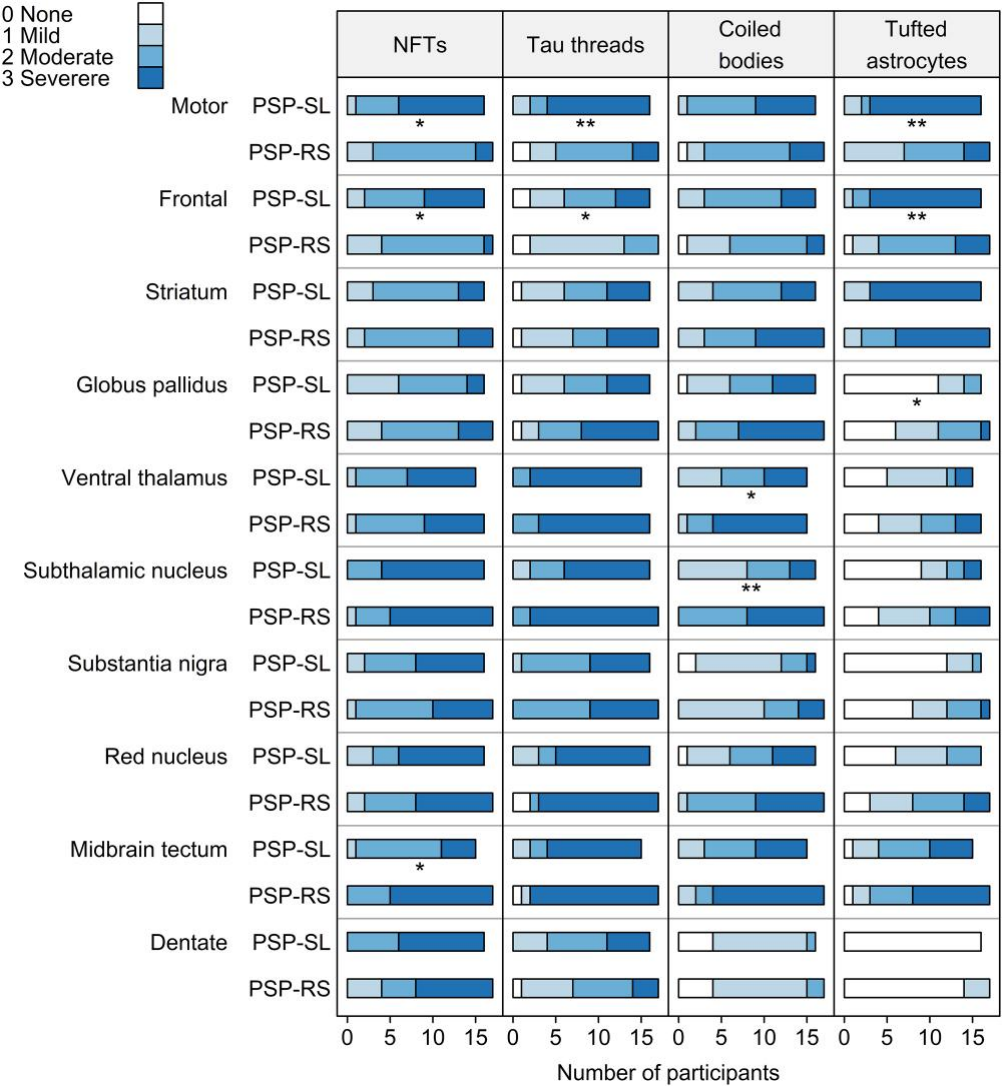
**Comparison of different tau lesion scores across ROIs in PSP-RS (*n* = 17) and PSP-SL (*n* = 16)**. Figure shows the proportion of cases in each group with each semi-quantitative tau lesion score. Statistical comparisons between PSP-RS and PSP-SL were performed using Wilcoxon rank sum test. *P < 0.05 uncorrected; **P < 0.05 after FDR correction for multiple comparisons. NFTs, neurofibrillary tangles.

Distribution of individual tau lesion scores (neurofibrillary tangle, neuropil threads, coiled bodies and tufted astrocytes) across groups is shown in Fig. 1. In the motor cortex, PSP-SL had higher threads and tufted astrocyte burden compared with PSP-RS, with most PSP-SL patients obtaining maximum scores in these lesion subtypes. The superior frontal cortex also showed more frequent tufted astrocytes in PSP-SL compared with PSP-RS. The burden of neurofibrillary tangles in motor and superior frontal cortices, and threads in superior frontal cortex, was also greater in PSP-SL, although these findings did not survive correction. Conversely, PSP-RS had a higher burden of coiled bodies in the subthalamic nucleus compared with PSP-SL. The burden of coiled bodies in the ventral thalamus, tufted astrocytes in the globus pallidus and neurofibrillary tangles in the midbrain was also greater in PSP-RS, although these findings did not survive correction.

### Neuroimaging differences across PSP groups

Comparisons of volumetric and DTI metrics between the PSP variants are displayed in Table 3. Smaller volumes of motor cortex were observed in PSP-SL, whilst the superior frontal cortex showed a similar degree of volume loss in both groups. PSP-RS showed smaller volume of the midbrain, as well as smaller volumes of the subthalamic nucleus and red nucleus, although the findings for these two regions did not survive correction. The other subcortical, brainstem and cerebellar regions were comparable between the two variants. In the DTI analysis, PSP-RS showed lower FA in both the midbrain and superior cerebellar peduncle, and greater MD in the midbrain, compared with PSP-SL. In cortical regions, MD was greater in the superior frontal cortex in PSP-SL compared with PSP-RS., although this finding did not survive correction.

**Table 3:** Comparison of volume *Z*-scores and DTI parameters across groups

	Volume *Z*-scores				Fractional anisotropy				Mean diffusivity			
	PSP-RS (*n* = 17)	PSP-SL (*n* = 16)	AUROC (95% CI)	*P*-value	PSP-RS (*n* = 11)	PSP-SL (*n* = 10)	AUROC (95% CI)	*P*-value	PSP-RS (*n* = 11)	PSP-SL (*n* = 10)	AUROC (95% CI)	*P*-value
**Motor cortex**	−0.95 [−1.15 to −0.17]	−1.93 [−2.26 to 0.909]	0.76 (0.56, 0.88)	**0**.01*	0.43 [0.41–0.44]	0.40 [0.38–0.43]	0.61 (0.36, 0.81)	0.44	759 [724–782]	795 [784–838]	0.76 (0.50, 0.90)	0.08
**Superior frontal cortex**	−1.56 [−2.15 to −1.32]	−1.93 [−2.37 to −1.23]	0.54 (0.35, 0.72)	0.71	0.41 [0.39–0.43]	0.38 [0.37–0.40]	0.72 (0.46, 0.88)	0.11	779 [761–817]	837 [811–885]	0.78 (0.52, 0.91)	**0.04**
**Striatum**	−1.39 [−2.06 to −0.459]	−1.04 [−1.44 to 0.288]	0.62 (0.42, 0.78)	0.25	-	-		-	771 [754–807]	824 [808–871]	0.66 (0.41, 0.84)	0.25
**Globus pallidus**	−0.57 [−0.66 to 0.003]	−0.52 [−0.89 to 0.33]	0.50 (0.32, 0.68)	0.99	-	-		-	800 [788–818]	801 [740–858]	0.51 (0.28, 0.73)	>0.99
**Ventral thalamus**	−0.64 [−1.18 to 0.20]	−1.06 [−1.64 to 0.59]	0.62 (0.42, 0.78)	0.19	-	-		-	765 [754–787]	789 [753–821]	0.60 (0.35, 0.80)	0.39
**Subthalamic nucleus**	−2.13 [−3.18 to −1.95]	−1.70 [−2.09 to 1.18]	0.76 (0.56, 0.88)	**0**.**03**	-	-		-	-	-		-
**Substantia nigra**	−2.53 [−3.38 to −2.14]	−2.18 [−3.03 to −1.50]	0.64 (0.44, 0.80)	0.18	-	-		-	-	-		-
**Red nucleus**	−3.26 [−4.52 to −2.72]	−2.52 [−3.10 to −2.22]	0.72 (0.52, 0.86)	**0**.**02**	-	-		-	-	-		-
**Midbrain**	−2.82 [−3.43 to −2.22]	−1.92 [−2.56 to −1.30]	0.78 (0.59, 0.90)	**0**.005*	0.42 [0.41–0.44]	0.46 [0.44–0.47]	0.89 (0.64, 0.97)	**0.002** *	798 [776–840]	715 [701–766]	0.87 (0.62, 0.96)	**0.004** *
**Dentate nucleus**	−1.91 [−3.03 to −0.87]	−1.67 [−2.31 to −1.21]	0.60 (0.41, 0.77)	0.31	-	-		-	-	-		-
**Superior cerebellar peduncle**	-	-		-	0.57 [0.55–0.60]	0.62 [0.61–0.66]	0.86 (0.61, 0.96)	**0.005** *	796 [772–805]	758 [748–796]	0.65 (0.40, 0.83)	0.28

Data are reported as median [Q1, Q3] or count (%).

Volume *Z*-scores were obtained by comparison with a group of controls matched by age, sex and education.

Threshold for significance was set at *P* < 0.05. Significant *P*-values are reported in bold.

AUROC, area under the receiver operator characteristic curve.

^*^ *P*-values significant after FDR correction.

### Correlations between tau burden and neuroimaging

**Figure 2:**
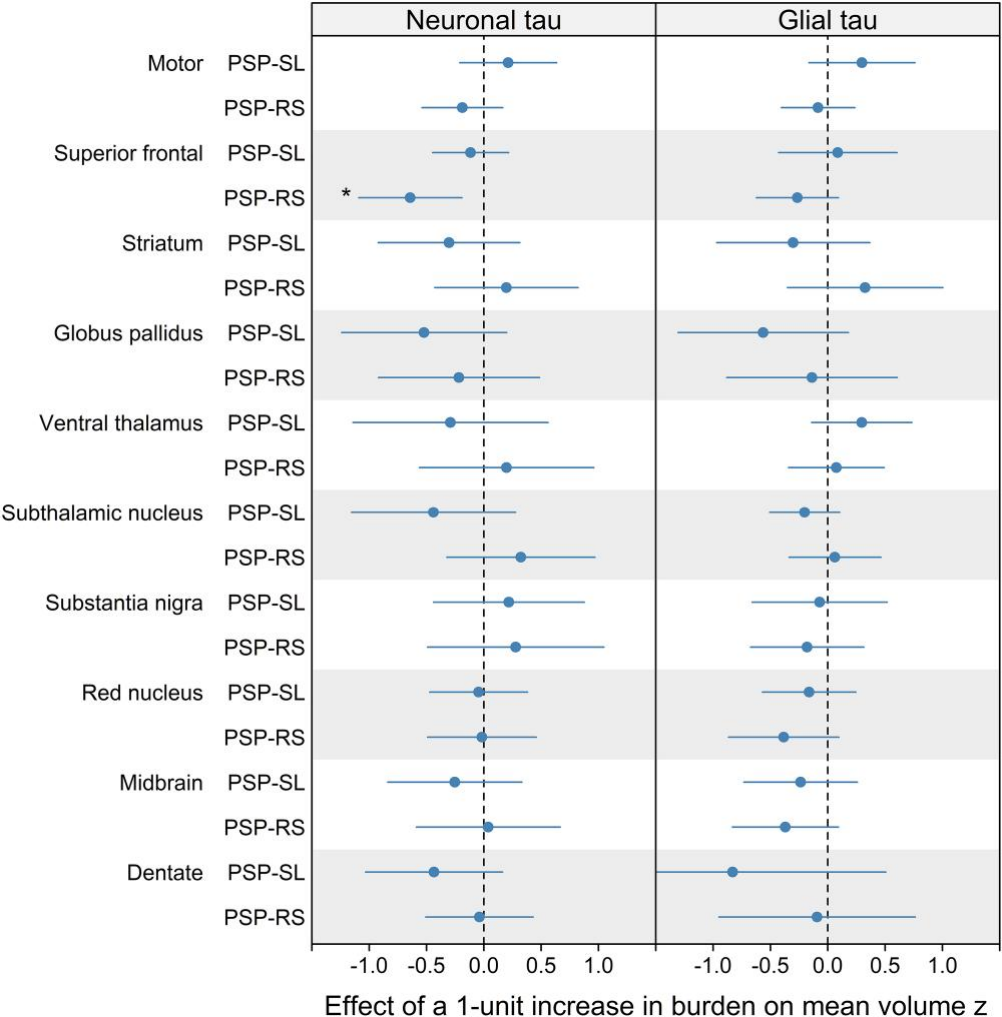
**Forest plots showing the relationship between volume and both neuronal and glial tau burden in PSP-RS (*n* = 17) and PSP-SL (*n* = 16)**. Forest plots show point estimates and corresponding 95% confidence interval for the effect of one-unit increase in the combined semi-quantitative scores for neuronal and glial tau on volume expressed in *Z*-scores. Statistical analysis performed with linear regression models, with volume as the response and the semi-quantitative tau burden as the predictors of interest, with age at death and time from MRI scan to death as covariates. Confidence intervals not crossing the line of null effect are considered significant. *P < 0.05 uncorrected (no findings survived FDR correction for multiple comparisons).

Figure 2 shows the estimated changes in volume associated with increases in neuronal and glial tau burden (all estimates, confidence intervals and *P*-values are shown in Supplementary Table 3). The only region that showed a relationship between tau and volume was the superior frontal cortex, with a one-unit increase in neuronal tau pathology being associated with a 0.64 decrease in *Z*-score converted volumes in the PSP-RS group (Supplementary Fig. 1), although this relationship did not survive correction. No relationship was observed between tau burden and volume in any region in PSP-SL.

**Figure 3:**
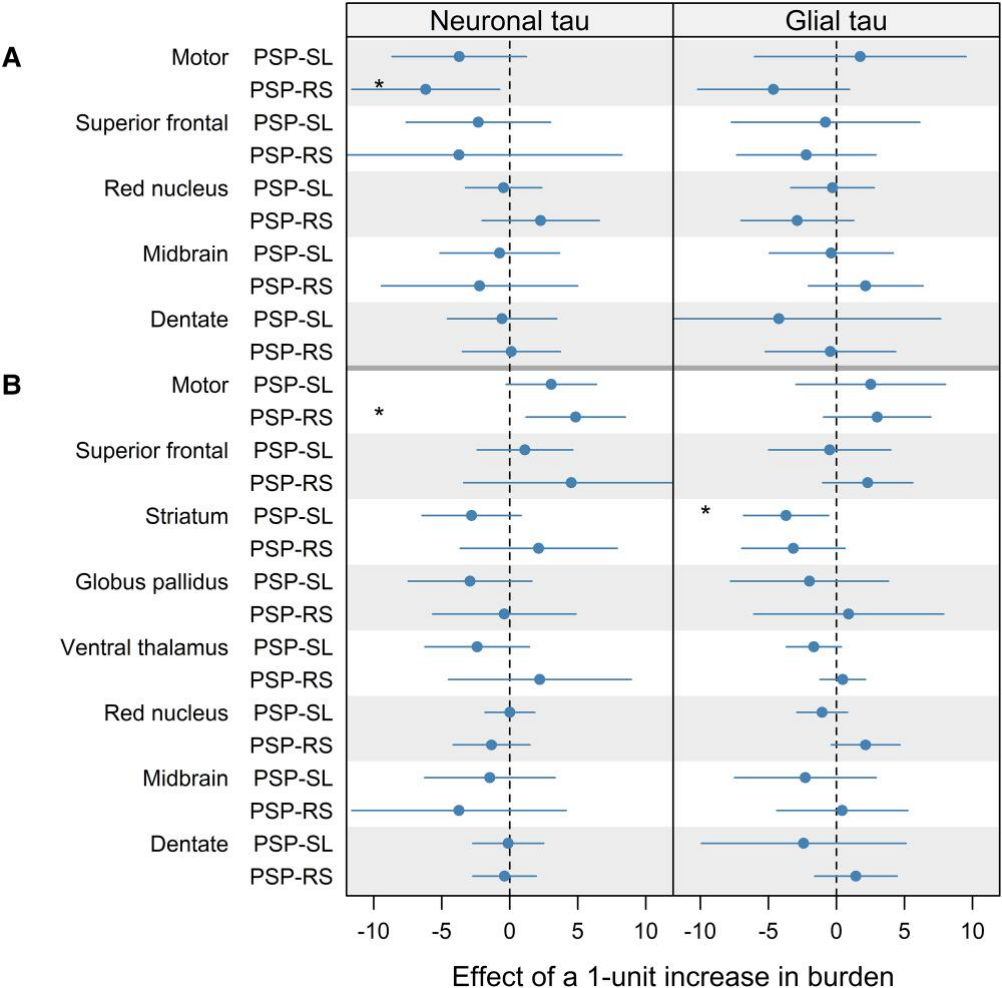
**Forest plots showing the relationship between neuronal and glial tau and DTI FA and MD in PSP-RS (*n* = 11) and PSP-SL (*n* = 10)**. Forest plots display point estimate and corresponding 95% confidence interval for the effect of one-unit increase in semi-quantitative tau pathology score on FA (**A**) and MD (**B**) indexes. Estimates are expressed in % change. Statistical analysis performed with linear regression models with DTI variables as the response and the semi-quantitative tau burden as the predictors of interest, with age at death and time from MRI scan to death as covariates. Confidence intervals not crossing line of null effect are considered significant. *P < 0.05 uncorrected (no findings survived FDR correction for multiple comparisons).

Regression coefficients for the effects of neuronal and glial tau burden on DTI metrics are displayed in Fig. 3 (all estimates, confidence intervals and *P*-values are shown in Supplementary Tables 4 and 5**).** A relationship was observed between FA and neuronal tau in the motor cortex in PSP-RS (Supplementary Fig. 1), with a one-unit increase in neuronal tau burden associated with a ∼8% decrease in FA in PSP-RS. MD was also associated with neuronal tau in the motor cortex in PSP-RS (Supplementary Fig. 1), with a one-unit increase in neuronal tau burden associated with a ∼6% increase in MD in the motor cortex. In PSP-SL, higher glial tau burden in the striatum was associated with ∼3% decreased MD (Supplementary Fig. 1). However, these relationships with DTI did not survive FDR correction.

## Discussion

In this study, we explored the neuropathological underpinnings of the PSP-RS and PSP-SL clinical presentations of PSP. We provided evidence that the regional neuropathological tau burden differs across these clinical variants likely explaining the differing clinical presentations. Somewhat surprisingly, tau burden did not consistently predict volume or DTI abnormalities across ROIs.

Comparison between neuropathological substrates of PSP-RS and PSP-SL confirmed more pathology in neocortical regions in PSP-SL. This helps to explain the prototypical speech and language manifestations observed early in this group. In both the motor cortex and superior frontal cortex, we found neuronal and glial tau was increased in PSP-SL compared with PSP-RS. These findings are compatible with previously described atypical forms of PSP first arising in frontal associative cortices^3,10^ and featuring the bulk of pathology in cortical regions. When assessing specific tau inclusions, the greatest difference between variants was observed in the burden of tau threads and tufted astrocytes in the motor cortex and tufted astrocytes in the superior frontal cortex, suggesting that these inclusions may be particularly important for the development of speech and language impairment. On imaging, PSP-SL showed smaller volumes of the motor cortex compared with PSP-RS, consistent with previous descriptions in clinical cohorts^24^ and with findings suggesting early involvement of posterior frontal regions in the cortical variants of PSP.^54^ Surprisingly, this was not the case in the superior frontal cortex, where there were comparable volumes across the two variants, despite the difference in the amount of tau pathology. We hypothesize this could be due to undersampling of the region pathologically, since the pathological region sampled is a relatively small coronal section of the lateral superior frontal gyrus, whilst the imaging ROI includes the whole superior frontal gyrus. This large ROI on neuroimaging may not allow us to discern very focal differences in volume loss between the two groups. Whilst smaller imaging ROIs could be generated, smaller ROIs are less likely to spatially overlap with the pathological ROIs across a large group of people since the pathological ROI is not selected with as great a precision from person to person as the automated MRI ROIs. Past studies have mapped apraxia of speech, the most common clinical presentation in our PSP-SL cohort, to very circumscribed cortical foci of atrophy of the bilateral premotor cortex,^19,55^ which our regional analysis may have failed to capture. Despite not having observed any volume difference in this ROI across groups, increased MD values support significantly more damage in the superior frontal cortex in PSP-SL compared with PSP-RS. This is in line with a previous study from our group, which reported better performance of DTI than volumetric MRI biomarkers in identifying structural damage related to tau pathology, particularly in neocortical areas.^28^

Given the more severe motor and oculomotor impairment seen amongst PSP-RS patients, we expected to find increased tau pathology in subcortical and brainstem regions compared with PSP-SL, as previously reported in another study.^10^ However, our analysis revealed similar neuronal tau burden in subcortical and brainstem regions in PSP-SL and PSP-RS, with both groups showing severe pathology. Greater glial tau burden was observed in these regions in PSP-RS, with the difference being mostly driven by coiled bodies. Our finding is in line with a recent study by Kovacs *et al*.,^46^ who reported PSP-RS and PSP-SL have the same level of neuronal and astroglial tau burden in the brainstem and subcortical regions, with more coiled bodies localized to the medulla oblongata and globus pallidus in PSP-RS. The subcortical region with the strongest difference in glial pathology in our cohort was the subthalamic nucleus. This concurred with the volume findings, where we found some evidence for smaller subthalamic volumes in PSP-RS compared with PSP-SL. The similarity in tau burden across some subcortical and brainstem regions may not be surprising; however, since patients with PSP-SL often develop the classic features of PSP-RS over time, most commonly meeting clinical criteria for PSP 6–7 years after onset.^17^ In this cohort, postural instability/falls, as well as supranuclear gaze palsy, developed in 44%, and akinesia developed in 88% of the PSP-SL patients by the last clinical evaluation. We hypothesize that the development of tau in subcortical and brainstem regions is related to this clinical progression.

Despite relatively similar tau burden, PSP-RS had more atrophy in the midbrain compared with PSP-SL, as already described in past papers,^19,24^ as well as greater MD and reduced FA in the midbrain, supporting previous papers.^25^ Indeed, midbrain atrophy has been traditionally considered as the most reliable imaging biomarker supporting a diagnosis of typical PSP,^56^ with the ‘hummingbird sign’,^57^ the most commonly used in clinical practice as an adjunct to neurological examination. Whilst past studies assessed midbrain volume early in the PSP-SL disease course, we show in this study that less midbrain atrophy compared with PSP-RS is still detectable closer to death. This indicates that atrophy in PSP-SL does not completely ‘catch up’ with PSP-RS, even at the latter stages of the disease. The mismatch between the degree of tau burden and volume differences across variants in the midbrain could be because the MRI was performed 2.6 years before death in the PSP-SL patients and it is likely that further atrophy will occur during this time. However, we did not observe any correlations between volume and tau burden in either PSP group even when correcting for time between MRI and death. The fact that tau burden across all four lesions was moderate–severe (score of 2–3) in the midbrain in most cases in both groups would have limited range in the data to observe correlations, however, since the maximum on our qualitative scale was reached. Future studies assessing quantitative tau burden will be needed to further assess these relationships, although these measurements also have inherent limitations. Another hypothesis for the mismatch is that tau pathology occurs later in the disease course in the midbrain in PSP-SL and due to the presumed lag between tau deposition and subsequent atrophy, midbrain volume has not decreased to the level observed in PSP-RS.

Tau burden did not consistently predict volume across ROIs. We only found a weak association of neuronal tau with volume loss in the superior frontal cortex amongst PSP-RS, but not amongst PSP-SL, supporting a causative role of tau in neurodegeneration, although this finding did not survive correction for multiple comparisons. A previous study has similarly suggested that neuronal, rather than glial, tau is particularly associated with neurodegeneration.^58^ The lack of an association in PSP-SL could be explained by PSP-SL having maxed out tau severity scores, which would not give enough range to estimate the putative presence of a correlation, in contrast with PSP-RS, which showed more within-group tau score variability. However, these findings need to be interpreted with caution, given the mismatch between superior frontal pathologic and imaging ROIs. We also found some evidence for associations between tau burden and DTI metrics in the motor cortex. In both PSP groups, there was a tendency for neuronal tau to predict a decrease in FA and an increase in MD. Curiously, there was weak evidence that increased glial burden was associated with decreased MD in the striatum in PSP-SL. This finding is very similar to our previous study where we found that this counter-intuitive finding was driven by astrocytic pathology,^28^ and supports the hypothesis of a protective role played by astrocytes against white matter structural damage. However, these results did not survive a correction for multiple comparisons, possibly because the smaller sample size in the DTI analysis. Hence, overall, there is weak evidence that tau is related to reduced volume and diffusivity in these PSP variants, although the lack of associations is likely to be largely due to challenges with this type of imaging–pathology analysis rather than the likelihood that tau is not related to neurodegeneration. Findings will also likely be strongly influenced by the cohort utilized and the range of pathological tau burden. We have previously found evidence that tau measured on PET is related to both local and distant volume loss and diffusivity changes in PSP.^59^ Future studies with larger cohorts and quantitative tau burden will be needed to further investigate these relationships.

Overall, the distribution of pathology and imaging differences allows us to hypothesize that the two PSP variants under study may start from distinct disease epicentres, with a neocortical epicentre in PSP-SL and a brainstem/subcortical epicentre in PSP-RS. Differences in the neocortex persisted until death, with greater tau burden in the motor cortex and smaller volumes close to death. However, early striking differences in midbrain volume may diminish over time with similar tau burden at death. Some of the subcortical differences, particularly the subthalamic nucleus and glial burden across regions, also persisted until death with greater tau burden in PSP-RS, although other regions showed similar tau burden and volumes close to death, such as the striatum and thalamus. There was evidence from the DTI measures that PSP-RS also still had greater involvement of the superior cerebellar peduncle close to death, although cerebellar dentate tau burden did not differ. These findings may suggest heterogeneity in the rate of progression of different regions within this PSP network of structures and that factors governing patterns of spread may be complicated. The concept that different variants of PSP have different early epicentres concurs with a recent study that used machine learning techniques to model disease progression on MRI in PSP.^54^ That study identified cortical and subcortical subtypes of PSP that had different early regional epicentres. However, in that study, the two variants had the same patterns of volume loss at later stages in the disease, which disagrees with our pathological and imaging findings. That study did not, however, assess autopsy findings and did not follow patients to death and instead used cross-sectional findings to draw conclusions regarding later disease stages. Nevertheless, both studies highlight how different PSP variants involve the same network of PSP-related regions, albeit to different degrees in some parts of the network.

Demographic differences were observed between the two PSP variants, with older age and longer disease duration in PSP-SL compared with PSP-RS. Long disease duration is an unavoidable feature of PSP-SL, since patients have relatively isolated speech and language features for many years before developing features of PSP. Disease duration differences may have influenced burden and MRI comparisons between these variants, although it is difficult to correct for such potential confounding effects when it is unknown if rate of disease spread may also differ across variants.^58^ Furthermore, it may not be sensible to try to model the pathological burden in PSP-SL early in the disease (e.g. 3 or 4 years from onset) since many patients do not have PSP features at this point in the disease.

One of the strengths of this study was undoubtedly the inclusion of autopsy-proven PSP cases that were all evaluated by one experienced board-certified neuropathologist. Other strengths include the fact that all patients in this study were enrolled and prospectively followed and underwent identical tests. Nevertheless, limitations include the already mentioned poor correspondence between the size of the pathological versus neuroimaging sampling of the ROI and the use of tau semi-quantitative scoring, which may not be the ideal way to perform correlation analyses, given its discrete and small range of values. Future studies will be needed to validate our findings using a quantitative approach to assess tau burden. We were unable to assess asymmetry in the relationships between tau burden and imaging findings in this study since the pathological analysis only sampled one hemisphere. Neurodegeneration and tau burden may be expected to be asymmetric, with greater involvement of the left hemisphere, in many of the PSP-SL patients. The left hemisphere was, however, sampled in most cases allowing us to capture the hemisphere with the greatest expected pathology. We were unable to match the hemisphere in two PSP-SL cases, which could have led to inaccurate imaging–pathology relationships in these cases. Ordinal predictors such as our tau burden measures can present challenges in regression analysis. However, we assessed both straight-line and restricted cubic spline fits and found that for regions that were significant for the straight-line fit, the restricted cubic spline model was providing similar estimates.

## Conclusion

We provide an in-depth characterization of the histological and neuroimaging differences between typical PSP-RS and PSP-SL. We demonstrated in a large sample that tau pathology impacts the neocortex in PSP-SL in comparison with PSP-RS, with similar involvement of many brainstem and subcortical structures. Our findings represent an important step forward in understanding the pathobiology of these variants of PSP, improving understanding of the pathological underpinnings of clinical progression in these patients and addressing the need for sound neuroimaging biomarkers for PSP diagnosis, although more research is needed. The pathological similarities between PSP-RS and PSP-SL, and overlapping clinical features later in the disease, may support the use of similar patient care and management strategies in both patient cohorts.

## Supplementary Material

fcae113_Supplementary_Data

## Data Availability

Data related to the findings presented in this paper are available from the corresponding author upon reasonable request.
