# Supplementary material for: Histologic tau lesions and magnetic resonance imaging biomarkers differ across two progressive supranuclear palsy variants
Source: Brain Commun. 2024 Apr 5;6(2):fcae113. doi: 10.1093/braincomms/fcae113 (PMC11040515; doi:10.1093/braincomms/fcae113)
Supplement: fcae113_Supplementary_Data [file fcae113_supplementary_data.docx]

**Supplementary Materials**

**Supplementary Table 1: Demographic features of the matched controls compared to the whole PSP cohort.**

|  | **Controls (n=33)** | **PSP cohort (n=33)** |
| --- | --- | --- |
| No. Females (%) | 12 (36%) | 12 (36%) |
| Education, years | 16 (14, 18) | 16 (14, 18) |
| Age at MRI | 74 (67, 77) | 74 (67, 77) |
| MRI manufacturer |  |  |
| GE | 21 (64%) | 21 (64%) |
| Siemens | 12 (36%) | 12 (36%) |

Data are reported as median [Q1, Q3] or count (%).

**Supplementary Table 2. PSP features present in PSP-RS and PSP-SL at last evaluation.**

|  | **PSP-RS (n=17)** | **PSP-SL (n=16)** |
| --- | --- | --- |
| Patients without oculomotor dysfunction (no 02 or 03) | 0 | 1 (6%) |
| Slowing of saccades only (02) | 1 (6%) | 8 (50%) |
| Supranuclear gaze palsy (03) | 16 (94%) | 7 (44%) |
| Postural instability/falls (P1 or P2) | 17 (100%) | 7 (44%) |
| Akinesia (A2 or A3) | 17 (100%) | 14 (88%) |
| Meets probable PSP-RS criteria | 17 (100%) | 3 (19%)* |

PSP-RS = PSP Richardson Syndrome; PSP-SL = PSP Speech and Language. The OPAC levels defined in the diagnostic criteria for PSP are provided. *Only three patients met criteria for PSP-RS since postural instability/falls must occur within three years of disease onset

**Supplementary Table 3. Regression models for effect of neuronal and glial tau lesions on volume change**

|  | **NEURONAL TAU** | | | | | | **GLIAL TAU** | | | | | |
| --- | --- | --- | --- | --- | --- | --- | --- | --- | --- | --- | --- | --- |
|  | **Pathology effect**  **p-value** | **Coefficient**  **PSP-RS** | **p-value** | **Coefficient**  **PSP-SL** | **p-value** | **Interaction term**  **p-value** | **Pathology effect**  **p-value** | **Coefficient**  **PSP-RS** | **p-value** | **Coefficient**  **PSP-SL** | **p-value** | **Interaction term**  **p-value** |
| **Superior Frontal** | **0.02** | −0.64  [−1.74, 2.18] | **0.008** | −0.12 [−1.80, 2.12] | 0.48 | **0.07** | 0.33 | −0.26 [−1.78, 2.14] | 0.15 | 0.08 [−1.71, 2.21] | 0.75 | 0.29 |
| **Motor** | 0.32 | −0.19  [−1.78, 2.14] | 0.30 | 0.21 [−1.75, 2.17] | 0.32 | 0.14 | 0.47 | −0.08 [−1.80, 2.12] | 0.61 | 0.26 [−1.73, 2.19] | 0.28 | 0.23 |
| **Striatum** | 0.47 | 0.21  [−1.66, 2.26] | 0.50 | −0.30 [−1.66, 2.26] | 0.34 | 0.23 | 0.34 | 0.35 [−1.63, 2.29] | 0.30 | −0.29 [−1.63, 2.29] | 0.39 | 0.15 |
| **GP** | 0.30 | −0.21  [−1.62, 2.30] | 0.54 | −0.53 [−1.61, 2.31] | 0.15 | 0.51 | 0.34 | −0.12 [−1.60, 2.32] | 0.75 | −0.54 [−1.60, 2.32] | 0.15 | 0.38 |
| **Thalamus** | 0.64 | 0.19  [−1.61, 2.31] | 0.59 | −0.30 [−1.57, 2.35] | 0.45 | 0.35 | 0.58 | 0.06 [−1.76, 2.16] | 0.76 | 0.21  [−1.75, 2.17] | 0.32 | 0.61 |
| **Subthalamic** | 0.22 | 0.32  [−1.64, 2.28] | 0.33 | −0.53 [−1.60, 2.32] | 0.15 | 0.09 | 0.30 | 0.05 [−1.76, 2.16] | 0.80 | −0.23  [−1.81, 2.11] | 0.14 | 0.25 |
| **Red nucleus** | 0.97 | −0.02  [−1.73, 2.19] | 0.93 | −0.05 [−1.75, 2.17] | 0.81 | 0.92 | 0.21 | −0.38 [−1.73, 2.19] | 0.11 | −0.16  [−1.76, 2.16] | 0.42 | 0.47 |
| **Midbrain** | 0.69 | 0.03  [−1.65, 2.27] | 0.93 | −0.25 [−1.67, 2.25] | 0.39 | 0.51 | 0.35 | −0.34 [−1.73, 2.19] | 0.15 | −0.04 [−1.75, 2.17] | 0.85 | 0.34 |
| **SN** | 0.66 | 0.28  [−1.59, 2.33] | 0.46 | −0.02 [−1.92, 2.00] | 0.69 | 0.85 | 0.75 | −0.18 [−1.72, 2.20] | 0.46 | −0.06 [−1.67, 2.25] | 0.82 | 0.75 |
| **Dentate** | 0.37 | −0.04  [−1.73, 2.19] | 0.87 | −0.42 [−1.67, 2.25] | 0.16 | 0.31 | 0.46 | −0.09 [−1.54, 2.38] | 0.84 | −0.82 [−1.31, 2.61] | 0.22 | 0.35 |

Estimated regression coefficients are reported with corresponding standard error in brackets. Significant p-values are highlighted in bold.

GP= globus pallidus; SN = substantia nigra

**Supplementary Table 4. Effects of neuronal and glial tau on FA change**

|  | **NEURONAL TAU** | | | | | | **GLIAL TAU** | | | | | |
| --- | --- | --- | --- | --- | --- | --- | --- | --- | --- | --- | --- | --- |
|  | **Pathology effect**  **p-value** | **Coefficient**  **PSP-RS** | **p-value** | **Coefficient**  **PSP-SL** | **p-value** | **Interaction**  **term**  **p-value** | **Pathology effect**  **p-value** | **Coefficient**  **PSP-RS** | **p-value** | **Coefficient**  **PSP-SL** | **p-value** | **Interaction term**  **p-value** |
| **Superior Frontal** | 0.55 | -3.54  [-14.62 – 8.98] | 0.54 | -2.15  [-7.31 – 3.29] | 0.40 | 0.83 | 0.66 | -2.16  [-7.10 – 3.04] | 0.38 | -0.58  [-7.32 – 6.66] | 0.86 | 0.70 |
| **Motor** | **0.02** | -7.48  [-13.34 – -1.22 | **0.02** | -4.68  [-9.37 – 0.26] | 0.06 | 0.43 | 0.28 | -4.56  [-10.10 – 1.33] | 0.12 | 0.52  [-7.54 – 9.28] | 0.90 | 0.31 |
| **Red nucleus** | 0.39 | 2.19  [-2.48 – 7.09] | 0.34 | -1.51  [-4.46 – 1.54] | 0.30 | 0.19 | 0.35 | -2.86  [-7.23 – 1.71] | 0.20 | -0.91  [-4.22 – 2.52] | 0.58 | 0.48 |
| **Midbrain** | 0.73 | -2.15  [-8.63 – 4.80] | 0.51 | -0.74  [-4.81 – 3.50] | 0.71 | 0.72 | 0.53 | 2.16  [-1.85 – 6.34] | 0.27 | -0.33  [-3.61 – 3.07] | 0.84 | 0.33 |
| **SN** | 0.96 | 0.11  [-3.81 – 4.18] | 0.96 | -0.57  [-4.91 – 3.96] | 0.79 | 0.80 | 0.72 | -0.43  [-5.58 – 5.00] | 0.86 | -4.74  [-16.52 – 8.69] | 0.44 | 0.52 |
| **Dentate** | 0.55 | -3.54  [-14.62 – 8.98] | 0.54 | -2.15  [-7.31 – 3.29] | 0.40 | 0.83 | 0.66 | -2.16  [-7.10 – 3.04] | 0.38 | -0.58  [-7.32 – 6.66] | 0.86 | 0.70 |

Regression coefficients represent % change in FA per unit of increase neuronal and glial pathology with corresponding 95% CI in brackets.

SN = substantia nigra

**Supplementary Table 5. Effect of neuronal and glial tau pathology on MD change**

|  | **NEURONAL TAU** | | | | | | **GLIAL TAU** | | | | | |
| --- | --- | --- | --- | --- | --- | --- | --- | --- | --- | --- | --- | --- |
|  | **Pathology effect**  **p-value** | **Coefficient**  **PSP-RS** | **p-value** | **Coefficient**  **PSP-SL** | **p-value** | **Interaction term**  **p-value** | **Pathology effect**  **p-value** | **Coefficient**  **PSP-RS** | **p-value** | **Coefficient**  **PSP-SL** | **p-value** | **Interaction term**  **p-value** |
| **Superior Frontal** | 0.33 | 4.60  [-3.15 – 13.09] | 0.23 | 1.31  [-2.08 – 4.81] | 0.43 | 0.44 | 0.35 | 2.33  [-1 – 5.65] | 0.16 | -0.15  [-4.50 – 4.39] | 0.94 | 0.37 |
| **Motor** | **0.01** | 5.55  [1.51 – 9.75] | **0.01** | 3.05  [-0.007 – 6.18] | 0.05 | 0.30 | 0.19 | 3.05  [-0.64 – 6.93] | 0.10 | 1.61  [-3.38 – 7.04] | 0.50 | 0.66 |
| **Striatum** | 0.32 | 2.22  [-4.21 – 9.09] | 0.49 | -2.37 [-6.29 – 1.71] | 0.23 | 0.19 | 0.07 | -3.25  [-7.41 – 1.01] | 0.12 | -3.34  [-6.75 – 0.20] | 0.06 | 0.98 |
| **GP** | 0.39 | -0.80  [-6.57 – 5.44] | 0.79 | -3.34  [-8.24 – 1.82] | 0.19 | 0.49 | 0.52 | 0.60  [ -6.9 – 8.82] | 0.86 | -3.44  [-9.52 – 3.03] | 0.27 | 0.41 |
| **Thalamus** | 0.22 | 2.42  [-4.30 – 9.63] | 0.46 | -3.15  [-6.85 – 0.70] | 0.10 | 0.17 | 0.52 | 0.47  [-1.39 – 2.33] | 0.60 | -1.09  [-3.30 – 1.18] | 0.32 | 0.27 |
| **Red nucleus** | 0.62 | -1.29  [-4.11 – 1.51] | 0.34 | 0.10  [-1.78 – 2.02] | 0.91 | 0.45 | 0.16 | 2.12  [-0.47 – 4.81] | 0.10 | -1  [-2.86 – 0.89] | 0.27 | *0.06* |
| **Midbrain** | 0.49 | -3.44  [-10.60 – 4.39] | 0.35 | -1.49  [-6.11 – 3.25] | 0.49 | 0.66 | 0.45 | 0.40  [-4.09 – 5.13] | 0.85 | -2.28  [-5.96 – 1.54] | 0.22 | 0.35 |
| **Dentate** | 0.94 | -0.40  [-2.66 – 2.02] | 0.74 | -0.20  [-2.86 – 2.43] | 0.86 | 0.93 | 0.51 | 1.44  [-1.63 – 4.60] | 0.34 | -2.53  [-9.69 – 5.19] | 0.48 | 0.33 |

Regression coefficients represent % change in MD per one-unit change in neuronal and glial tau. Significant p-values are highlighted in bold.

GP= globus pallidus; SN = substantia nigra

**Supplementary Figure 1: Scatterplots showing the significant relationships between tau burden and the neuroimaging metrics.** The straight-line fits are from linear regression models while the curves are fit using linear regression but modeling the effect of neuronal or glial tau burden using a restricted cubic spline. FA = fractional anisotropy; MD = mean diffusivity

**
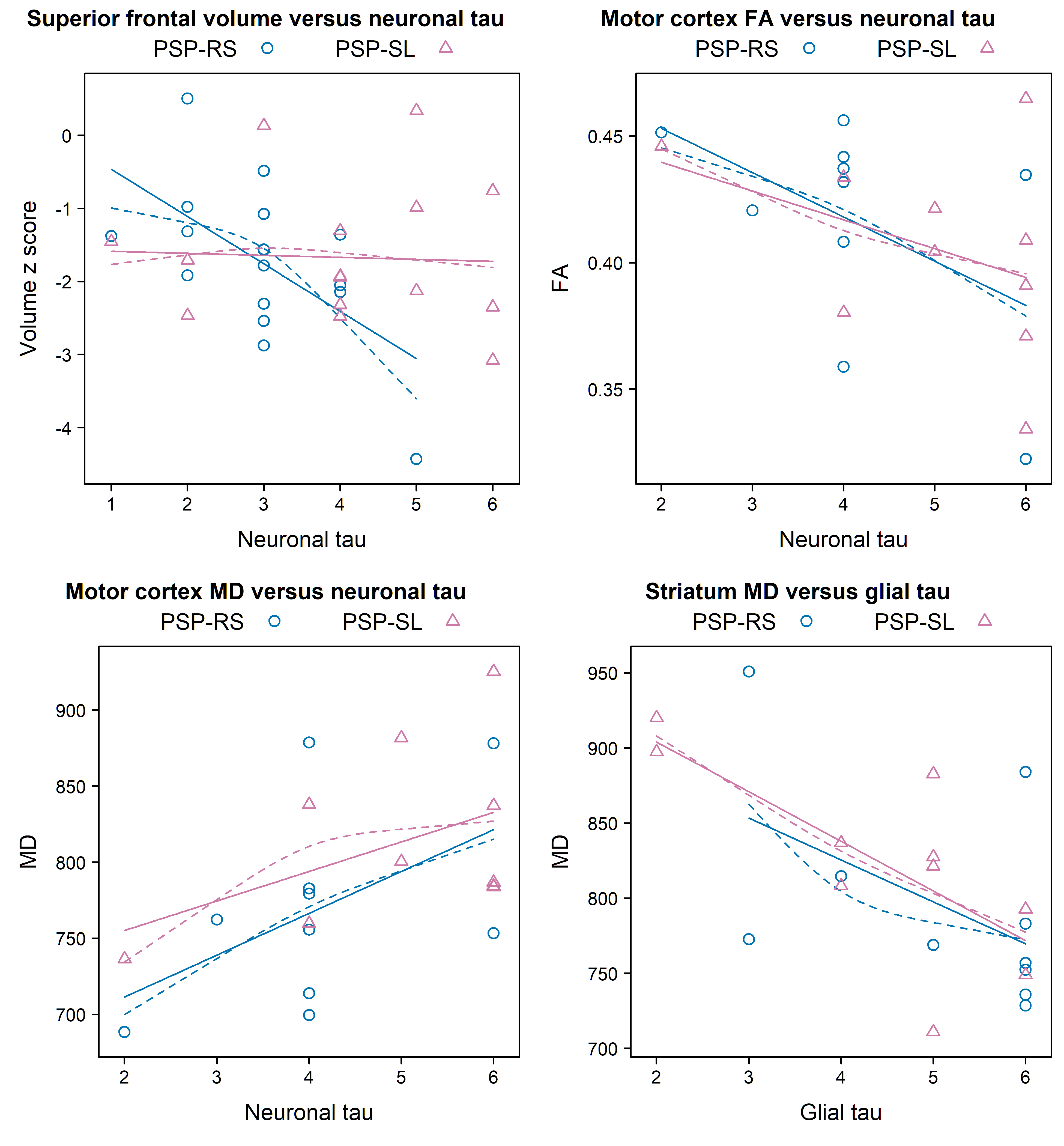
**
